# Mesenchymal stem cell-derived exosomes in cancer therapy resistance: recent advances and therapeutic potential

**DOI:** 10.1186/s12943-022-01650-5

**Published:** 2022-09-13

**Authors:** Zhengjun Lin, Yanlin Wu, Yiting Xu, Guoqing Li, Zhihong Li, Tang Liu

**Affiliations:** 1 Department of Orthopedics, The Second Xiangya Hospital of Central South University, Changsha, 410011 Hunan People’s Republic of China; 2 Xiangya School of Medicine, Central South University, Changsha, 410013 Hunan People’s Republic of China

**Keywords:** Mesenchymal stem cell, Exosome, Therapy resistance, Cancer

## Abstract

Mesenchymal stem cells (MSCs) are multipotent stromal cells that can be obtained from various human tissues and organs. They can differentiate into a wide range of cell types, including osteoblasts, adipocytes and chondrocytes, thus exhibiting great potential in regenerative medicine. Numerous studies have indicated that MSCs play critical roles in cancer biology. The crosstalk between tumour cells and MSCs has been found to regulate many tumour behaviours, such as proliferation, metastasis and epithelial-mesenchymal transition (EMT). Multiple lines of evidence have demonstrated that MSCs can secrete exosomes that can modulate the tumour microenvironment and play important roles in tumour development. Notably, very recent works have shown that mesenchymal stem cell-derived exosomes (MSC-derived exosomes) are critically involved in cancer resistance to chemotherapy agents, targeted-therapy drugs, radiotherapy and immunotherapy. In this review, we systematically summarized the emerging roles and detailed molecular mechanisms of MSC-derived exosomes in mediating cancer therapy resistance, thus providing novel insights into the clinical applications of MSC-derived exosomes in cancer management.

## Background

Cancer is a major disease and the leading cause of mortality worldwide [1]. Although several treatment approaches, including surgery, chemotherapy, radiotherapy, targeted therapy, and immunotherapy have been developed and effectively improved patients’ prognoses, both intrinsic and acquired therapy resistance contribute to the reduction of therapeutic effectiveness, thus resulting in poor outcomes of cancer patients [2, 3]. The mechanisms of cancer therapy resistance are complicated and multifaceted, including decreased uptake and/or increased efflux of drugs, the inhibition of apoptosis-related signalling pathways, activation of cancer stem cells (CSCs), enhanced DNA damage repair, loss of cell cycle control, as well as physical barriers preventing therapeutic drugs from entering the tissue and exerting their effects [4–6]. Nevertheless, the detailed mechanisms underlying anticancer therapy resistance are still unclear. Identifying the key mediators of cancer therapy resistance could help us to understand the development of therapy resistance, and these mediators play considerable roles in the prediction and reversion of anticancer therapy resistance.

Mesenchymal stem cells (MSCs) are multipotent stem cells that can self-renew and differentiate into multilineage cells [7]. MSCs can be obtained from a variety of human tissues and organs, such as the bone marrow, fat tissue, brain, lung, and pancreas [7]. Moreover, emerging evidence has highlighted the regulatory roles of MSCs in both physiological and pathological conditions [8]. MSCs have the innate affinity to home to tumour sites and can modulate multiple biological processes related to cancer, such as metastasis, angiogenesis and epithelial-mesenchymal transition (EMT) [9, 10]. Notably, MSCs can secrete exosomes that can interact with diverse recipient cells to affect diverse biological behaviours of targeted cells, thereby modulating physiological homeostasis and/or the progression of human diseases [11]. Exosomes are nanosized membrane vesicles 40–160 nm in diameter that can be secreted by diverse cell types [12, 13]. Exosomes comprise complex contents, such as nucleic acids including DNA, mRNAs, and noncoding RNAs (ncRNAs), lipids, and various proteins [14, 15]. The major function of exosomes is modulating intercellular communication between diverse cell types by transferring multiple molecules, which mediate various cellular processes in both physiological and pathological conditions [16, 17]. Notably, numerous works have found that MSC-derived exosomes can function as critical modulators in the tumour microenvironment, and mediate the pathogenesis of various human cancers [18, 19]. Interestingly, MSC-derived exosomes have been found to play an essential role in cancer therapy resistance, including chemoresistance, targeted therapy resistance, radiotherapy resistance and immunotherapy resistance [20–22]. Furthermore, MSC-derived exosomes have been exploited as a novel therapeutic strategy in antitumour treatment due to their various advantages, such as biocompatibility, promising potential for modification, and lack of cytotoxicity [20–22]. For instance, MSC-derived exosomes show great potential for the delivery of anticancer agents because they can enhance their efficacy with low toxicity. Additionally, a great number of investigations have indicated that MSC-derived exosomes transfected with synthetic miRNAs can effectively enhance the sensitivity of cancer cells to chemotherapeutic drugs [18, 20].

In this review, we performed a systematic review of the pivotal roles of MSC-derived exosomes in mediating cancer therapy resistance as well as the complex mechanisms by which MSC-derived exosomes enhance or weaken anticancer-therapy resistance. Meanwhile, we also discussed the clinical applications of MSC-derived exosomes as novel therapeutic strategies for enhancing drug sensitivity, drug delivery vehicles and radiation-induced damage repair in cancer management.

## Overview of MSC-derived exosomes

### Biogenesis of exosomes

**Fig. 1:**
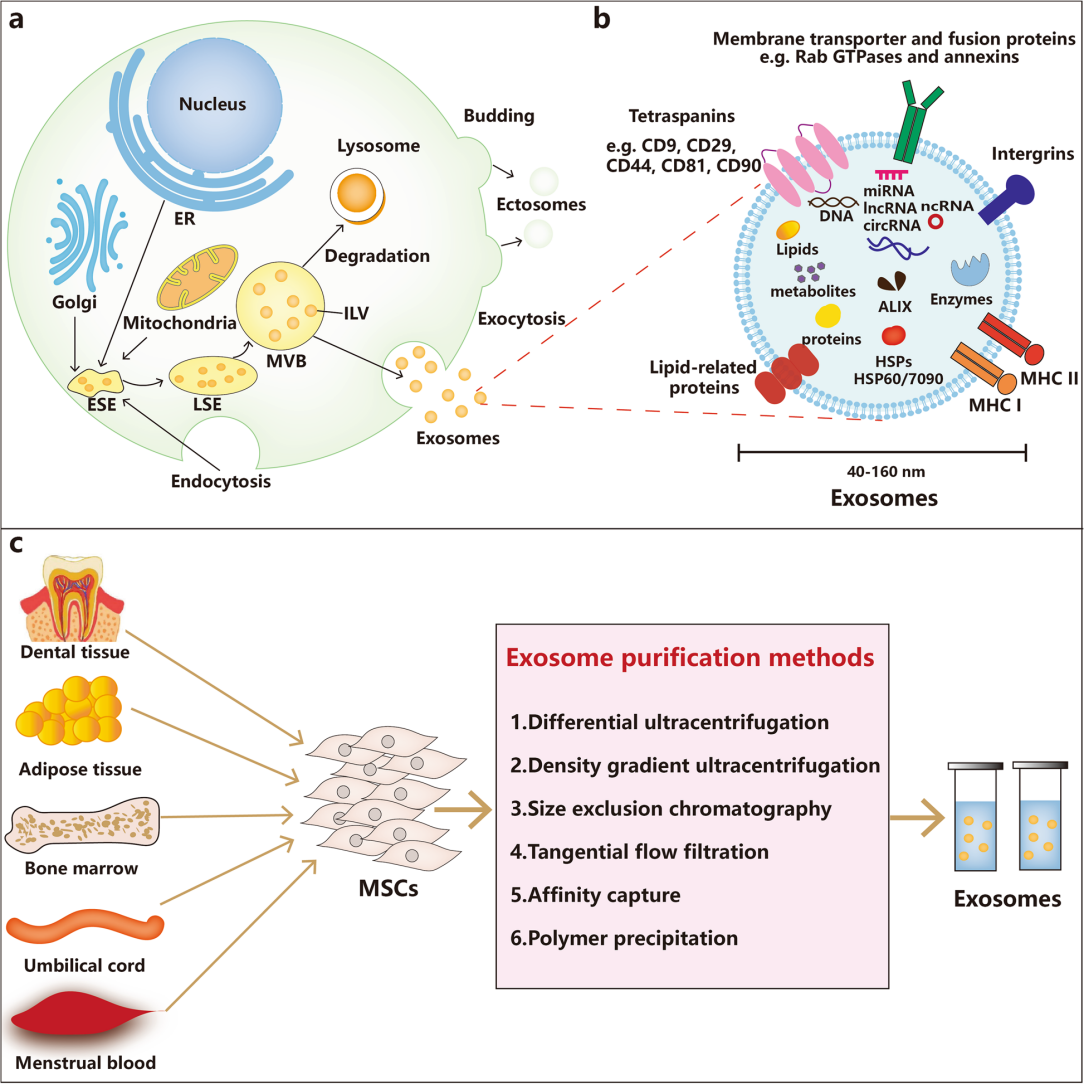
Overview of MSC-derived exosomes. **A** Biogenesis of exosomes. The biogenesis of exosomes comprises endocytosis, MVB formation, and exosome secretion into the extracellular microenvironment through merging with the plasma membrane. **B** Characteristics and contents of exosomes. **C** Exosome purification methods

Exosomes are of endosomal origin and derived from lipid raft microdomains in the plasma membrane, with a diameter ranging in size from 40 to 160 nm and a density of 1.15–1.19 g/mL in sucrose gradients [23–25]. Exosomes are characterized as having a round morphology with heterogeneous sizes under a transmission electron microscope [26]. Exosomes are derived from a sequential process, mainly including the dual invagination of the plasma membrane, and the formation of intracellular multivesicular bodies (MVBs) that contain intraluminal vesicles (ILVs) (Fig. 1a) [27]. The biogenesis of exosomes starts with the invagination of primary endocytic vesicles encysting proteins from the cell surface and extracellular environments, which form the early-sorting endosomes (ESEs). Some ESEs are returned to the plasma membrane by endosome recycling, whereas other ESEs can induce the formation of late sorting endosomes (LSEs), and invagination of LSEs gives rise to ILVs [27]. This process contributes to the modification of cargos in the future resulting exosomes and the subsequent formation of MVBs. While some MVBs will be degraded by autophagosomes or lysosomes, others can fuse with the plasma membrane and then release ILVs into the extracellular environment as exosomes [28].

A variety of proteins have been found to modulate the biogenesis of exosomes. Endosomal-sorting complex required for transport (ESCRT) proteins (ESCRT-0, ESCRT-I, ESCRT-II and ESCRT-III) are recognized as crucial mediators of ILV formation [29]. They can sort protein cargoes, induce membrane budding and sever membrane necks from their inner face with accessory proteins including Alix, VPS4 and VTA1 [30]. During the process of ILV biogenesis, protein cargoes captured into ILVs are principally characterized by ubiquitin, and the ESCRT-0 complex recognizes and obtains ubiquitinated proteins in the late endosome membrane [31, 32]. Then, cargoes are handed over to ESCRT-I, followed by the recruitment of ESCRT-II, which comprises a ubiquitin-binding and PtdIns3P-binding GLUE domain [29]. ESCRT-I and ESCRT-II complexes have strong recognition domains with high affinity for ubiquitinated substrates, and are responsible for shapping the membrane into buds, but it is still unknown whether ESCRT-I and ESCRT-II bind ubiquitylated cargoes sequentially or coordinately [33, 34]. ESCRT-III separates the vesicles from the cytoplasmic membrane to form MVBs, and promotes the deubiquitination of cargo proteins by deubiquitylating enzymes (DUBs) before ILV formation [14, 35]. Finally, ATPase induces the membrane scission and vacuolar protein sorting 4 (VPS4) participates in recycling all ESCRT molecules [36, 37]. The ubiquitinated proteins are critically involved in ESCRT-dependent cargo sorting and ILV formation, and E3 ubiquitin ligases and deubiquitylating enzymes have been identified to modulate the localization and functions of ESCRTs [38, 39]. In addition to ESCRT-dependent biogenesis of exosomes, recent reports have emphasized the importance of ESCRT-independent pathways involving ceramides, ADP ribosylation factor 6 (ARF6), and tetraspanin proteins (CD9, CD81 and CD82) in exosome biogenesis [40]. There also exists a non-MVB-specific pathway that regulates ILV formation through protein–protein interactions, in which syntenin-1 can bind to the cytosolic domain of syndecan-1 to recruit Alix in synergy with ESCRTs [41]. In terms of the fusion of MVBs with the plasma membrane and exosome secretion, RAB family small GTPase proteins, including Rab27a, Rab27b, Rab35 and Rab7, as well as membrane fusion soluble N-ethylmaleimide-sensitive factor attachment protein receptor (SNARE) complex proteins have been demonstrated to play critical roles in vesicular sorting and trafficking towards secretory organelles [42]. Interestingly, it has been reported that the mechanism of exosome secretion modulated by various RAB family proteins is dependent on cellular types. For instance, Rab27a and Rab27b are involved in controlling exosome release in HeLa cells, while exosome release in MCF-7 breast cancer cells is mediated by Rab7 [43]. Although progress in understanding the numerous proteins controlling the origin and biogenesis of exosomes has been achieved, the underlying mechanisms by which these proteins exert precise rate-limiting effects on exosome biogenesis warrant further in-depth exploration, especially in vivo research.

### Contents of exosomes

Exosomes carry a variety of substances, including proteins, lipids, nucleic acids (DNA, mRNAs, and ncRNAs) and metabolites (Fig. 1b) [18]. There are thousands of exosome cargos, such as proteins, mRNAs, and miRNAs, according to the ExoCarta database (http://exocarta.org/) [44]. These cargos are critically involved in regulating diverse biological processes in both physiological and pathological conditions, and can be employed as biomarkers and effective therapeutic targets for human diseases. Over 1600 proteins participating in different biological functions, such as signal transduction, structural dynamics and metabolism modulation, are carried in exosomes [23]. Multiple exosomal proteins have been found to be associated with membrane transport and fusion, such as RAB GTPases, ESCRTs, heat shock proteins (HSPs) such as Hsp60, Hsp70, and Hsp90 and the accessory factors Alix and VPS4 [23, 45, 46]. Tetraspanins including CD9, CD63, CD81, CD82, and Tspan8, are the most common membrane proteins in exosomes, and have been found to facilitate the packaging of specific cargos into exosomes [47]. Notably, several exosomal proteins that are nonspecific molecules are usually regarded as markers for the identification of exosomes, such as CD9, CD63, CD81, HSP70 and Alix [14, 48]. Lipids are also a key component in exosomes, and play an indispensable role in exosome biogenesis, shape maintenance and homeostasis regulation in recipient cells [14]. For example, lysobisphosphatidic acid and ceramide are crucial mediators during exosome release from the cytoplasm [49, 50]. In addition, growing evidence has shown that ceramide participates in the regulation of autophagy, which may influence MVB homeostasis indirectly [51]. Moreover, the lipid composition of target cells has been found to change after fusing with exosomes, which subsequently affects the homeostasis of exosomes in recipient cells [14]. Notably, nucleic acids, especially RNAs make up the most significant fraction of functional components in exosomes and significantly facilitate the roles of exosomes as critical modulators of intracellular communication and diverse signalling pathways [52]. Exosomal RNA packaging is a specific process, as indicated by the preferential accumulation of certain exosomal RNAs that can be transferred to recipient cells to exert their effects [43]. RNAs in exosomes can modulate the biological behaviours of recipient cells in various ways, and exosomal RNAs in different body fluids can function as diagnostic and prognostic biomarkers in various human diseases [53]. In addition to proteins, lipids and RNAs, exosomes also carry several types of DNA, including single-stranded DNA (ssDNA), double stranded DNA (dsDNA) and mitochondrial DNA (mtDNA). KRAS and p53 mutations can be identified in genomic DNA from exosomal DNA for pancreatic cancer prediction [54]. The transfer of exosomal DNA into recipient cells can also endow recipient cells with diverse biological functions. For example, topotecan-treated cancer cell-derived exosomal DNA can trigger the activation of dendritic cells and subsequent CD8+ T-cell activation through the CGAS-STING signalling pathway [55].

### Isolation of exosomes

In general, exosomes can be isolated from various body fluids, such as blood, urine, and synovial fluid, and conditioned cell culture media. Various techniques have been utilized for exosome isolation, such as differential ultracentrifugation, density-gradient ultracentrifugation, and tangential flow filtration (Fig. 1c) [56]. Differential high-speed ultracentrifugation is the most widely utilized and traditional method due to its simple protocol, efficiency and high purity [57]. Additionally, it has been found that density-gradient ultracentrifugation can isolate exosomes with a higher purity by isolating particles by layers of biocompatible medium with different densities, in comparison with classic ultracentrifugation [58]. However, all these methods, including density-gradient ultracentrifugation, size exclusion chromatography, and tangential flow filtration, have the same limitation in that they fail to distinguish particles with overlapping ranges, such as exosomes and microvesicles (extracellular vesicles with similar properties to exosomes) [20]. Notably, affinity capture can well distinguish a wide range of exosomes with high purity by surface markers of extracellular vesicles with the capture molecules attached to different carriers instead of depending on the density or size of extracellular vesicles; however, it has the disadvantage of low yield [20, 59]. Many novel techniques have emerged in recent years, including polyethylene glycol-based low-speed centrifugation, antibody- and filter-based enrichment methods, methods incorporating acoustics and microfluidics. In addition, many commercial kits have been put into use for exosome isolation. However, their isolation effectiveness has yet to be fully assessed [58, 60–62]. Although there is currently no consensus on a “gold standard” method for exosome isolation, it is recommended to utilize more than one technique combined for optimal outcomes in exosome isolation [63].

### Characteristics of MSC-derived exosomes

MSCs have been demonstrated to be the most prolific producers of mass exosomes in humans [64]. MSC-derived exosomes were first investigated in the myocardial ischemia/reperfusion injury in vivo in 2010, followed by numerous works focusing on the roles of MSC-derived exosomes in a variety of diseases [65]. MSC-derived exosomes have the same morphological characteristics and isolation and storage methods as exosomes from other sources [19]. In terms of the identification of exosomes, MSC-derived exosomes not only express common surface biomarkers such as CD81 and CD9, but also express MSC surface markers, such as CD29, CD44, CD73 and CD90. MSC-derived exosomes are also enriched in diverse contents similar to exosomes from other cellular sources [66]. A previous study identified 730 functional proteins in bone marrow mesenchymal stem cell (BMSC)-derived exosomes, which play important roles in regulating the cellular growth, proliferation, migration and morphogenesis capacities of MSCs [67]. RNAs also make up critical components of MSC-derived exosomes. It has been found that MSC-derived exosomes are mainly enriched in short RNAs (< 300 nt), whereas 28S and 18S RNAs are not visible by utilizing ethidium bromide staining [66]. The enrichment of miRNAs in MSC-derived exosomes has been extensively investigated. It has been reported that exosomal miRNAs from MSCs are predominantly in the precursor form, and an ample amount of passenger miRNA has been found in MSC-derived exosomes [68]. Furthermore, comparative analysis of MSC-derived exosomal miRNAs with miRNAs in MSCs have found that 106 miRNAs in MSCs were not detected in MSC-derived exosomes, indicating that the packaging of miRNAs into exosomes is not a random but a regulated process [69]. The mechanisms by which exosomes selectively package miRNAs remain largely unknown. Previous studies have confirmed the critical correlation between RNAs and RNA-binding proteins (RBPs) outside cells, indicative of the importance of RBPs in the transfer and maintenance of RNAs in the extracellular space [70–72]. Emerging evidence has indicated that various RBPs are critically involved in sorting miRNAs into exosomes, making RBPs critical candidates for the fate and function of exosomal miRNAs [71, 73]. Notably, very recent work has demonstrated that special sorting sequences of miRNAs are required to determine their secretion in small extracellular vesicles (EXO-motifs) or cellular retention (CELL-motifs) [74]. Moreover, Alyref and Fus are two important RBPs involved in miRNA sequence recognition and the packaging of miRNA in exosomes [74]. Multiple lines of evidence have identified that the biological functions of MSC-derived exosomes are similar to those of MSCs, such as tissue regeneration, immune regulatory effects, and anti-inflammatory effects [75]. Notably, the unique characteristics of MSC-derived exosomes, including small size, long circulation half-life, low immunogenicity, excellent penetration capability, and ideal biocompatibility, make them promising candidates for the treatment of diverse human diseases, especially as newly-developed tools to be exploited in anticancer therapy [66].

## MSC-derived exosomes in cancer therapy resistance

**Fig. 2:**
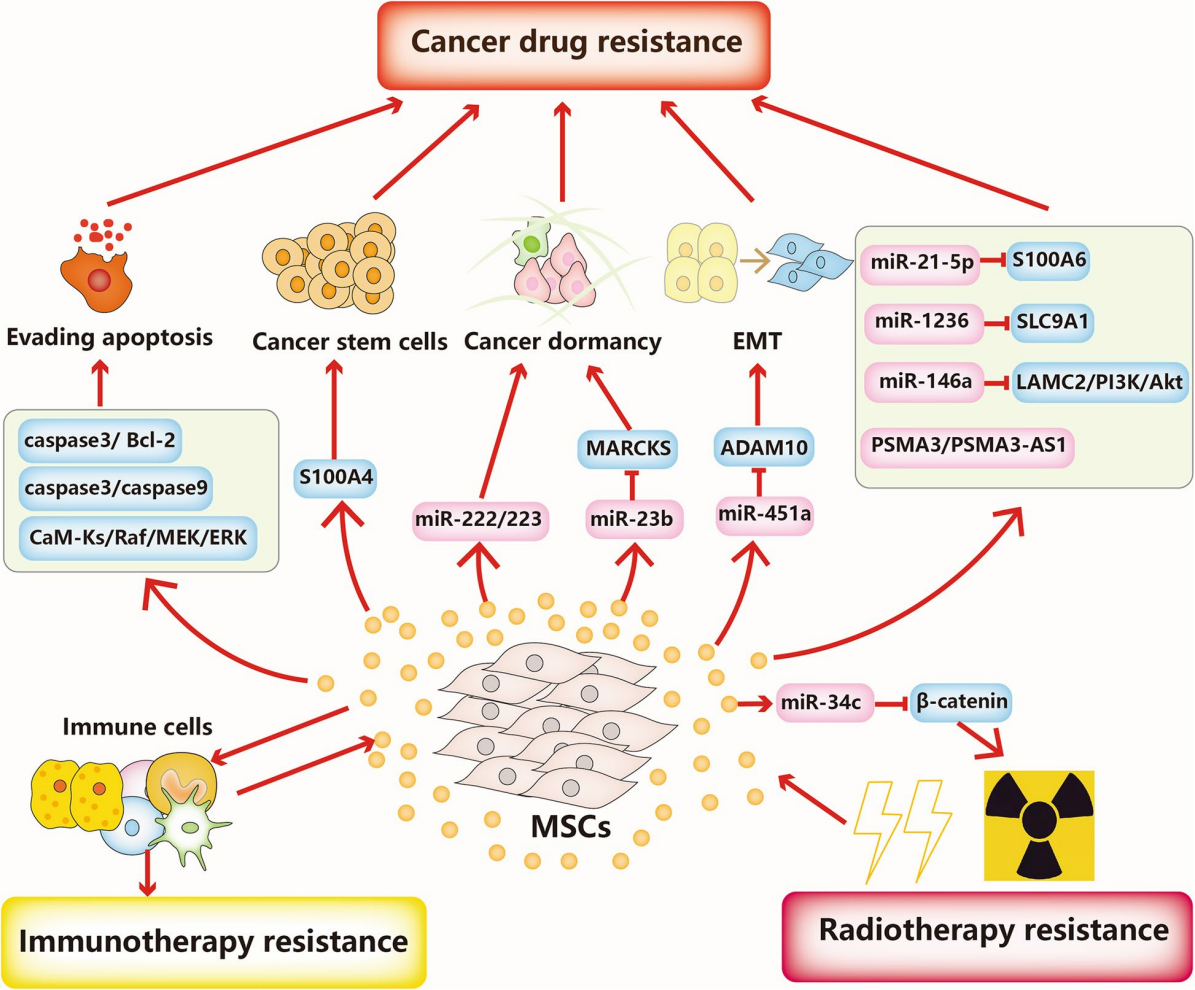
MSC-derived exosomes in cancer therapy resistance. MSC-derived exosomes participate in cancer drug resistance, immunotherapy and radiotherapy resistance through complex mechanisms, including evasion of apoptosis, modulation of cancer stem cells, and regulation of cancer dormancy

Therapeutic resistance is a daunting challenge in achieving cures in patients with cancer. Cancer therapy resistance can be categorized into primary resistance and acquired resistance. Primary resistance means that tumour cells are initially resistant to standard therapy due to genetic or phenotypic alterations, while acquired resistance occurs after the initial successful therapeutic responses [76]. Hence, elucidating the detailed mechanisms involved in cancer therapy resistance is critical for developing optimal therapeutic strategies and improving the prognosis of cancer patients. Critical roles played by MSC-derived exosomes in multiple aspects of cancer progression, particularly therapy resistance have been increasingly demonstrated. The involvement of MSC-derived exosomes in cancer resistance to chemotherapy, targeted-therapy, immunotherapy and radiotherapy is summarized below (Fig. 2).

### MSC-derived exosomes in chemotherapy resistance

Currently, chemotherapy is the first-line therapeutic strategy for a series of malignancies. Multiple chemotherapeutic agents with different properties and targets have been effectively applied to improve the clinical outcomes of patients with advanced cancers, such as platinum drugs, adriamycin (ADR), and 5-fluorouracil (5-FU) [77, 78]. However, the development of multidrug resistance (MDR) has remained the major challenge for the success of chemotherapy [79]. Multiple lines of evidence have demonstrated that MSC-derived exosomes are deeply involved in cancer chemotherapy resistance because they directly deliver functional proteins and RNAs (Table 1). Apoptosis avoidance has been identified as one of the major causes of chemoresistance, and MSC-derived exosomes can modulate apoptosis-related proteins to mediate chemoresistance. It has been found that MSC-derived exosomes enhance the resistance to 5-FU in gastric cancer both in vitro and in vivo. Mechanistically, MSC-derived exosomes prevent 5-FU-induced apoptosis by activating the CaM-Ks/Raf/MEK/ERK signalling cascade and MDR associated proteins by transferring functional proteins [80]. Tumour dormancy means that tumour cells can remain in the G0 phase of the cell cycle at the metastatic site after the resection of the primary tumour. Tumour dormancy is critically involved in cancer recurrence, metastasis and chemoresistance, and breast cancer cells exhibit particularly extensive tumour dormancy [81]. There is overwhelming evidence indicating that breast cancer recurrence and chemoresistance are correlated with the prolonged dormancy and successful survival of tumour cells in the bone marrow microenvironment. MSC-derived exosomes induced by breast cancer cells promoted breast cancer dormancy by transferring miR-222/223 in vitro and in vivo, and this dormancy was linked to carboplatin resistance. Furthermore, MSC-derived exosomes delivering antagomiRs enhanced the sensitivity to carboplatin and prevented cellular dormancy in breast cancer [22]. MSCs derived from human umbilical cord (hUCMSCs) are known for their promising self-renewal and proliferation capacities and their multipotent ability to differentiate into diverse cell lineages of the three germ layers [82]. hUCMSCs can also secrete diverse biologically active molecules through exosomes to interact with other cells, such as immune cells, thereby showing promising therapeutic potential in human diseases [82]. The antitumour effects of hUCMSCs have been confirmed in several neoplasms, such as breast cancer, lung cancer and osteosarcoma [83, 84]. Notably, however, hUCMSCs have been found to transdifferentiate into cancer-associated mesenchymal stem cells in tumour microenvironment, and have the capability to facilitate cancer progression and metastasis [85, 86]. Therefore, it is still largely unclear whether hUCMSCs promote or inhibit tumour progression, and the roles of hUCMSC-derived exosomes in cancer initiation and development are also uncertain and controversial. It has been found that hUCMSC-derived exosomes can promote dormancy initiation and confer resistance to conventional chemotherapy by inducing the expression of MMP-2 and ecto-5′-nucleotidase by transferring miRNAs in metastatic breast cancer [87]. Nevertheless, several studies have indicated hUCMSC-derived exosomes can inhibit breast cancer progression through complex mechanisms and deliver different cargoes. More studies are needed to clarify the controversial findings regarding the functions of hUCMSC-derived exosomes during breast cancer progression. It has been found that BMSC-derived exosomes can promote dormancy and docetaxel resistance in a bone marrow–metastatic human breast cancer cell line (BM2). Mechanistically, remarkable upregulation of miR-23b was detected in BMSC-derived exosomes compared with adult fibroblasts, and BMSC-derived exosomal miR-23b enhanced dormancy phenotypes and docetaxel resistance in BM2 cells by suppressing the expression of MARCKS, which encodes a protein that facilitates cell cycling and motility [88]. Similarly, MSCs-secreted exosomes have been found to promote doxorubicin (DOX) resistance by inducing S100A6 expression in MDA-MB-231 breast cancer cells. Upregulation of miR-21-5p has been detected in both MSCs and MSC-secreted exosomes after exposure to DOX, and MSCs-secreted exosomes could potentiate DOX resistance via miR-21-5p-mediated induction of S100A6 expression in breast cancer in vitro and in vivo [89]. CSCs, characterized by self-renewal and differentiation capacities and high tumorigenic potency, can differentiate into various subpopulations of cells within tumours [90]. It has been suggested that MSC-derived exosomes can regulate CSCs to mediate cancer therapy resistance. Exosomes from BMSCs have been found to upregulate stemness genes, such as *OCT4*, *NANOG* and *SOX2*, and promote the stemness of acute myeloid leukaemia (AML) cells. In addition, BMSC-derived exosomes can reduce the sensitivity to Ara-C by stimulating the expression of S100A4, a typical member of the S100 family of calcium-binding proteins in AML cells. Furthermore, knockdown of S100A4 reversed chemoresistance of AML cells [21].

**Table 1:** MSC-derived exosomes in chemotherapy resistance

Exosome source	Cancer type	Method	Key cargo	Genes and pathways	Drug	Drug resistance	Ref
hBMSCs	Gastric cancer	In vitro and in vivo		CaM-Ks/Raf/MEK/ERK	5-FU	↑	[80]
hBMSCs	Breast cancer	In vitro and in vivo	miR-222/223		Carboplatin	↑	[22]
hUMSCs	Breast cancer	In vitro		MMP-2 and ecto-5′-nucleotidase	Conventional chemotherapy	↑	[87]
hBMSCs	Breast cancer	In vitro	miR-23b	MARCKS	DOX	↑	[88]
hBMSCs	Breast cancer	In vitro	miR-21-5p	S100A6	DOX	↑	[89]
hBMSCs	Acute myeloid leukemia	In vitro		OCT4, NANOG and SOX2	Ara-C	↑	[21]
ADMSCs	Breast cancer	In vitro	miR-1236	Wnt/β-Catenin	DDP	↓	[91]
hUCMSCs	Ovarian cancer	In vitro	miR-146a	LAMC2/PI3K/Akt	DOX	↓	[92]
hUCMSCs	Hepatocellular carcinoma	In vitro	miR-451a	ADAM10	PTX	↓	[93]

In contrast, a number of studies have identified that MSC-derived exosomes can enhance chemosensitivity in various cancers. Adipose-derived mesenchymal stem cell (ADMSC)-derived exosomes have been reported to can impair cisplatin (DDP) resistance in both parental and DDP-resistant breast cancer cell lines. Mechanistic investigation demonstrated that ADMSC-derived exosomes enhanced sensitivity to DDP by downregulating SLC9A1 via miR-1236 in breast cancer. Furthermore, overexpression of SLC9A1 reversed the inhibitory effects of ADMSC-derived exosomal miR-1236 on DDP resistance by activating the Wnt/β-Catenin signalling pathway in breast cancer [91]. Exosomal miR-146a from hUCMSCs has been reported to reverse the resistance of ovarian cancer cells to docetaxel and taxane by modulating the expression of LAMC2 and the PI3K/Akt signalling pathway in vitro [92]. EMT is a biological process, in which cells lose their epithelial characteristics, such as apical-basal polarity and cell junctions, and transform into a mesenchymal phenotype [94]. Increasing evidence has confirmed that cancer cells can undergo EMT to acquire chemoresistance in various cancer types, indicating inhibiting that EMT is a feasible strategy for reversing chemoresistance [94]. Xu et al. confirmed that hUCMSC-derived exosomal miR-451a could inhibit the EMT of hepatocellular carcinoma Hep3B and SMMC-7721 cell lines through the inhibition of ADAM10, thus increasing the paclitaxel (PTX) sensitivity of hepatocellular carcinoma cells in vitro [93].

### MSC-derived exosomes in targeted therapy resistance

Recent advances in the understanding of the molecular biology of cancer development have given rise to numerous targeted-therapy drugs. Multiple targeted therapy drugs have been applied in the treatment of diverse cancers, such as cetuximab and panitumumab targeting the epidermal growth factor receptor (EGFR), and trastuzumab targeting HER-2 [95, 96]. Although these targeted-therapy drugs have been found to effectively improve the prognosis of cancer patients, the inevitable emergence of targeted-therapy resistance is a major hurdle for the long-term survival of patients [97]. Emerging evidence has indicated that MSC-derived exosomes critically participate in mediating targeted-therapy resistance (Table 2). For instance, Viola et al. have indicated that alterations in the contents of BMSC-derived exosomes were significantly correlated with tyrosine kinase inhibitor (TKI) resistance in AML [98]. Human BMSC (hBMSC)-derived exosomes have been reported to inhibit cellular proliferation via transferring miR-15a in chronic myeloid leukaemia (CML) in vitro. In addition, hBMSC-derived exosomes have also been confirmed to be involved in CML targeted-therapy resistance. hBMSC-derived exosomes can enhance cellular apoptosis resistance to TKIs by silencing caspase3 and Bcl-2 both in vitro and in vivo [99]. The proteasome inhibitor (PI) bortezomib has shown impressive efficacy in the treatment of patients with multiple myeloma [100]. However, its therapeutic effectiveness is highly impaired by drug resistance and the mechanism underlying PI resistance has not been completely revealed. A recent study suggested that MSC-derived exosomes play a critical role in multiple myeloma PI resistance. Furthermore, researchers identified that PSMA3 and lnc PSMA3-AS1 which were markedly upregulated in bortezomib-resistant samples in comparison to bortezomib-sensitive samples, were present in MSC-derived exosomes in multiple myeloma. Mechanistically, MSC-derived exosomes conferred bortezomib resistance by transferring PSMA3 and lnc PSMA3-AS1 to multiple myeloma cells, and lnc PSMA3-AS1 impaired the sensitivity of multiple myeloma cells to bortezomib by upregulating PSMA3 expression by forming an RNA duplex with PSMA3-AS1 pre-mRNA in vitro. Further in vivo studies confirmed that PSMA3-AS1 siRNA efficiently enhanced carfilzomib sensitivity in multiple myeloma xenografts, thereby identifying the therapeutic potential of exosomal PSMA3-AS1 from MSCs [101]. Conversely, MSC-derived exosomes can also reverse cancer targeted therapy resistance. Imatinib (IM), a TKI targeting the BCR-ABL oncoprotein, is a front-line therapeutic strategy for patients with early-stage CML [102]. However, approximately 20% CML patients develop IM resistance, resulting in unfavourable long-term survival. hUCMSC-derived exosomes have been found to enhance the sensitivity of K562 cells to IM through inhibition of proliferation and induction of apoptosis. Mechanistically, the combination of hUCMSC-derived exosomes with IM activated caspase-9 and caspase-3 in vitro in CML [103].

**Table 2:** MSC-derived exosomes in targeted therapy resistance

Exosome source	Cancer type	Method	Key cargo	Genes and pathways	Drugs	Drug resistance	Ref
BMSCs	AML	In vitro			TKIs	↑	[98]
hBMSCs	CML	In vivo and vitro		Caspase3 and Bcl-2	TKIs	↑	[99]
hBMSCs	Multiple myeloma	In vitro and in vivo	PSMA3 and Psma3-as1		Bortezomib	↑	[101]
hUMSCs	CML	In vitro		Caspase-9 and Caspase-3	IM	↓	[103]

### MSC-derived exosomes in radiotherapy resistance

Radiation therapy is also a critical component of cancer therapeutic strategies, and has been widely utilized in diverse cancer types, such as prostate cancer, lung cancer and head and neck cancer [104]. It has been reported that approximately 50% of patients with cancer receive radiation therapy during their treatment, and radiation therapy accounts for approximately 40% of curative treatment for cancer patients [105]. However, cancer patients often develop radioresistance via complex mechanisms, resulting in limited effectiveness and cancer metastasis and recurrence. Emerging evidence has demonstrated that radiation therapy can affect the contents and secretion of exosomes, and radiation-derived exosomes can confer radioresistance and facilitate radiation-induced bystander effects [106]. For example, exosomal miR-340-5p from hypoxic oesophageal squamous cell carcinoma (OSCC) cells potentiated radioresistance by preventing radiation-induced apoptosis and promoting DNA damage repair by directly targeting KLF10 [107]. Notably, MSC-derived exosomes also participate in mediating cancer radiotherapy resistance. miR-34c, which is downregulated in both nasopharyngeal carcinoma tissues and cell lines, has been found to impair radioresistance by promoting radiotherapy-induced cell apoptosis by slicing β-catenin in vitro and in vivo. Furthermore, miR-34c-overexpressing exosomes derived from MSCs drastically facilitated radiation-induced cellular apoptosis in nasopharyngeal carcinoma [108]. In addition, MSC-derived exosomes could enhance the effectiveness of radiotherapy in inhibiting tumour growth and metastasis in a melanoma mouse model, thus suggesting the utility of combining radiotherapy with MSC-derived exosomes in the treatment of cancer patients [109].

### MSC-derived exosomes in immunotherapy resistance

Recent breakthroughs in exploring the tumour immune microenvironment have opened new avenues for the development of immunotherapies [110]. Immunotherapy, which aims to kill cancer cells by modulating the host immune system, has been applied in the clinical treatment along with surgery, chemotherapy and radiotherapy in cancers such as melanoma [111]. Currently, the commonly used immunotherapeutic agents include immune checkpoint inhibitors, such as nivolumab and pembrolizumab (anti-PD-1), and ipilimumab and tremelimumab (anti-CTLA-4), chimeric antigen receptor (CAR) T cells and monoclonal antibodies [111–115]. Despite the unprecedented durable response rates of cancer patients to immunotherapies, a large portion of patients do not respond to immunotherapies due to primary, adaptive or acquired resistance [116]. Exosomes can play an immunoregulatory role by modulating the functions of different types of immune cells such as natural killer (NK) cells, T- and B-lymphocytes in the tumour immune microenvironment. Moreover, exosomes can transfer diverse cellular components and express molecules such as PD-L1 to stimulate or suppress the tumour immune response [117]. For example, hepatocellular carcinoma-derived exosomal circUHRF1 has been confirmed to inhibit the functions of NK cells by regulating the miR-449c-5p/TIM-3 pathway and can impair the sensitivity of hepatocellular carcinoma cells to anti-PD1 therapy [118]. In melanoma, it has been found that exosomal PD-L1 levels are associated with the tumour burden and immunotherapy sensitivity after patients receive PD-1 inhibitors, indicating the potential role of exosomal PD-L1 as a predictive biomarker for anti-PD-1 therapy. Notably, ongoing studies indicate that MSC-derived exosomes also critically participate in immunomodulation, mainly by regulating the functions of immune cells and altering the secretion of inflammatory factors, such as TNF-α and IL-1β [119]. MSC-derived exosomes can facilitate the differentiation of monocytic myeloid-derived suppressor cells (M-MDSCs) into highly immunosuppressive M2-polarized macrophages. In addition, MSC-derived exosomes can impair protective antitumour immunity through the upregulation of PD-L1 in myeloid cells and the downregulation of PD-1 in T cells in breast cancer in vivo [120]. In pancreatic ductal adenocarcinoma, BMSC-derived exosomes carrying galectin-9 siRNA and oxaliplatin (OXA) prodrug can induce immunogenic cell death (ICD), and reverse the suppressive tumour immune microenvironment, for example, inhibiting M2 macrophage polarization and the recruitment of cytotoxic T lymphocytes, thus enhancing immunotherapy effectiveness in vitro and in vivo [121]. Future studies are needed to elucidate whether MSC-derived exosomes play an immune-suppressive or immune-promoting role in the tumour microenvironment, as well as their functions in mediating cancer immunotherapy resistance.

## Therapeutic potential of MSC-derived exosomes in cancer

**Fig. 3:**
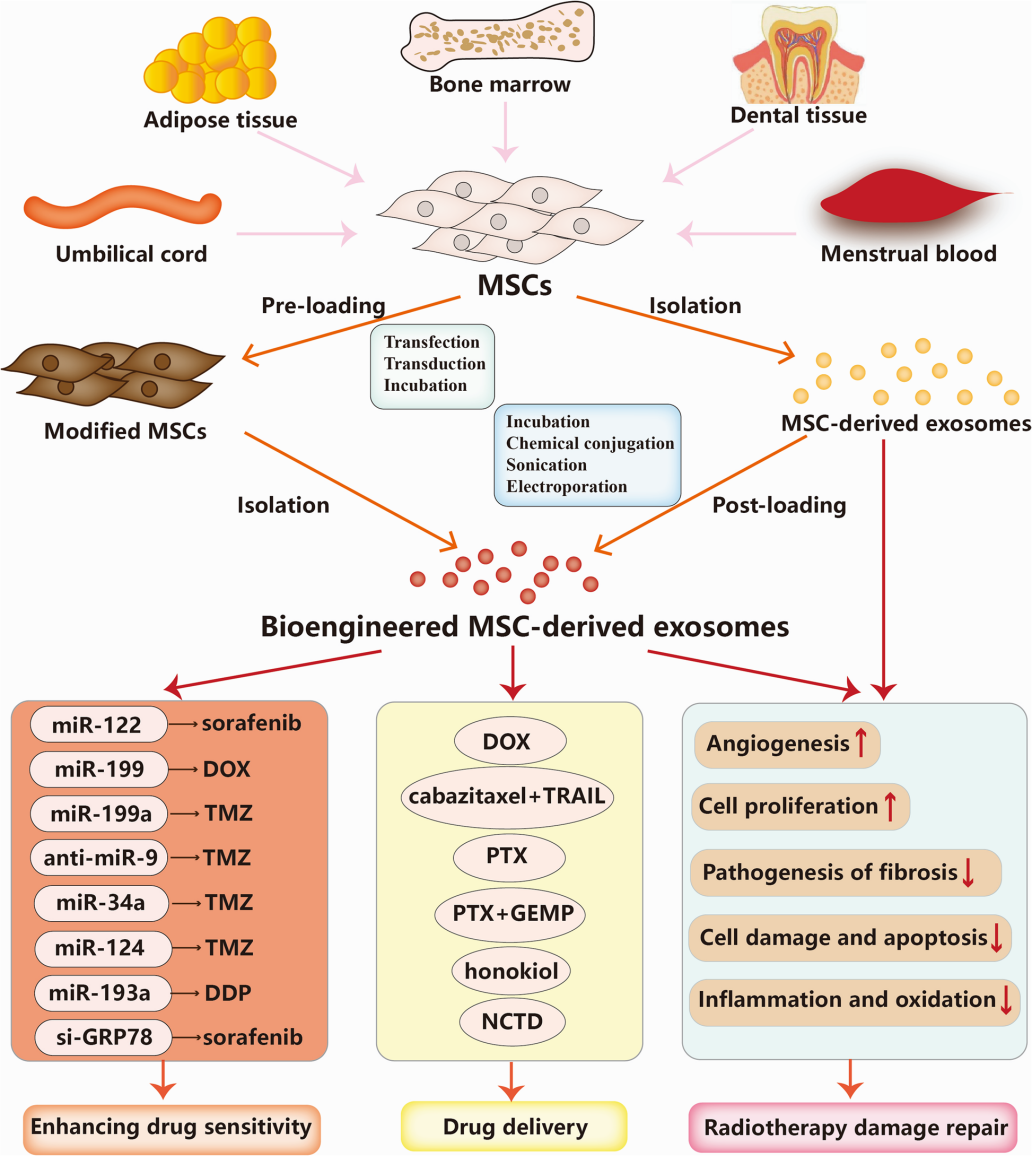
Applications of MSC-derived exosomes in cancer therapy. MSC-derived exosomes can be employed to deliver functional RNAs including miRNAs and siRNA to enhance the drug sensitivity, and deliver anticancer drugs such as chemotherapeutic agents. Moreover, MSC-derived exosomes are capable of repairing radiotherapy-induced damage through complex mechanisms, including promotion of cellular proliferation, promotion of angiogenesis capability, inhibition of inflammation and oxidation, and reduction of the pathogenesis of fibrosis

In the previous sections, we summarized the detailed mechanisms by which MSC-derived exosomes intensify or weaken cancer therapy resistance. Based on these mechanisms, in this section, we will discuss the promising potential of MSC-derived exosomes as a novel therapeutic strategy for enhancing therapeutic sensitivity, delivering various anticancer cargoes, and repairing radiotherapy-induced damage in cancer treatment (Fig. 3).

### MSC-derived exosomes for enhancing drug sensitivity

Recent progress in understanding the roles of MSC-derived exosomes in mediating cancer therapy resistance has highlighted the promising potential of MSC-derived exosome-based therapy for enhancing therapeutic sensitivity. Because of their natural intercellular communication function, strong tumour tropism, low immunogenicity, low toxicity, biodegradable characteristics, and capability to escape from clearance and cross biological barriers, MSC-derived exosomes have emerged as promising carriers of various biomolecules and chemical agents in cancer treatment. Bioengineered MSC-derived exosomes can encapsulate desired therapeutic cargoes, such as miRNAs, proteins and drugs. There are two main anti-cancer cargo loading approaches, including preloading (before exosome isolation) and post-loading (after exosome isolation). It has been observed that MSCs transfected with synthetic miRNAs can enhance the chemosensitivity of cancer cells by transferring specific miRNAs via exosomes. For instance, ADMSCs transfected with miR-122, which can effectively transfer miR-122 to hepatocellular carcinoma cells and have been found to sensitize hepatocellular carcinoma cells to 5-FU and sorafenib by negatively regulating the expression of miR-122 target genes. Furthermore, intratumour injection of ADMSC-derived miR-122-carrying exosomes can significantly enhance the antitumour efficacy of sorafenib and inhibit tumour growth in hepatocellular carcinoma in vivo [122]. Similarly, exosomes derived from miR-199-modified ADMSCs can improve sensitivity to DOX via the inhibition of the mTOR signalling pathway in hepatocellular carcinoma in vitro and in vivo [123]. In glioma, miR-199a, a downregulated miRNA in both glioma tissues and cells, has been found to inhibit the proliferation, invasion and migration of U251 cells in vitro. Furthermore, miR-199a-overexpressing MSC-derived exosomes inhibited glioma progression and enhanced sensitivity to temozolomide (TMZ) by suppressing AGAP2 expression in vitro and in vivo [124]. Exosomes from MSCs-labelled with anti-miR-9-Cy5 can transfer anti-miR-9 to weaken the TMZ resistance of glioblastoma cells. Mechanistically, anti-miR-9 delivered by MSC-derived exosomes can promote caspase activity and induce cell death in response to TMZ by suppressing the expression of the drug transporter MDR1 [125]. Likewise, it has been found that hBMSC-secreted exosomes overexpressing miR-34a can increase the sensitivity to TMZ, and inhibit the proliferation, migration and invasion of glioblastoma cells by downregulating MYCN both in vitro and in vivo [126]. Wharton’s jelly-derived MSC (WJ-MSC)-derived exosomes transfected with miR-124 have been confirmed to sensitize glioblastoma cells to TMZ and inhibit glioblastoma cell proliferation and migration of by directly targeting CDK6 in vitro [127]. It has been observed that miR-193a expression was downregulated whereas LRRC1 expression was upregulated in both DDP-resistant NSCLC tissues and cells, and BMSC-derived exosomes could inhibit NSCLC progression through upregulating miR-193a and downregulating LRRC1 in vitro and in vivo. Furthermore, BMSC-derived exosomes transfected with miR-193a mimic impaired DDP resistance and inhibited proliferation, migration and invasion by inhibiting LRRC expression in NSCLC [128]. Additionally, MSC-derived exosomes-delivered small interfering RNA (siRNA) is another novel therapeutic strategy for enhancing drug sensitivity in the treatment of many cancers. Grp78, which is upregulated in multiple cancer types, have been reported to confer sorafenib resistance in hepatocellular carcinoma. Exosomes derived from GRP7-siRNA-modified BMSCs can sensitize hepatocellular carcinoma cells to sorafenib, and combination of si-GRP78-modified BMSC-derived exosomes and sorafenib can suppress the growth and invasion of the hepatocellular carcinoma cells in vitro [129].

### MSC-derived exosomes for drug delivery

MSC-derived exosomes possess broad application prospects for delivering chemotherapeutic agents in cancer treatment and can effectively improve the inhibitory effects on tumour growth and selectivity for targeting tumour sites of the chemotherapeutic agents. Compared with conventional chemotherapy. Besides, MSC-derived exosomes can easily permeate through various physical barriers at a high speed, such as the blood–brain barrier (BBB), and transcytosis is the main underlying mechanism, thus helping drugs reach therapeutic concentrations [130]. In drug-resistant oral squamous cell carcinoma MSC-derived exosomes can deliver cabazitaxel/tumour necrosis factor-related apoptosis-inducing ligand (TRAIL) combinations and enhance cabazitaxel-induced apoptosis by inhibiting the PI3K/Akt/mTOR signalling pathway in vitro and vivo [131]. It has been found that MSC-derived exosomes carrying DOX can inhibit osteosarcoma cell growth with higher effectiveness and lower cytotoxicity than free DOX in vitro [132]. Similarly, Bagheri et al. reported that MSC-derived exosomes carrying DOX could be transported to MUC1-positive colorectal cancer cells with high efficiency in vitro. Further in vivo studies found that DOX carried in MSC-derived exosomes could highly accumulate in tumour site and remarkedly inhibit tumour growth with faster liver clearance than free DOX [133]. It has been found that mouse BMSCs could be loaded with PTX through exposure to very-high-dose PTX in vitro. Then, these BMSCs can secrete exosomes containing a high amount of PTX, which exert strong anti-proliferative effects on pancreatic cancer cells [134]. Similarly, PTX-loaded MSC-derived exosomes have been found to effectively inhibit tumour growth and distant organs metastasis with a 1000-fold reduced PTX amount compared with the application of free PTX [135]. Recent research has found that BMSC-derived exosomes loaded with PTX and gemcitabine monophosphate (GEMP), an intermediate product of gemcitabine metabolism, exhibited superior homing and penetrating abilities both in vitro and in vivo in pancreatic cancer. Meanwhile, both the in vitro and in vivo antitumour efficacy of BMSC-derived exosomes loaded with PTX and GEMP were significantly high. These findings indicated that BMSC-derived exosomes loaded with PTX and GEMP may play an important role in overcoming chemoresistance and penetrating pathological barriers, and could function well as a promising strategy for targeted therapy in pancreatic cancer [136]. Honokiol is a pleiotropic compound that has been specified as a novel antitumour agent in various cancer types [137]. Kanchanapally et al. loaded MSC-derived exosomes with honokiol by utilizing the sonication method, and the results showed that MSC-derived exosomes-loaded honokiol had superior antitumour effects than the free honokiol resulting from the efficient cellular uptake [138]. Norcantharidin, is a demethylated derivative of cantharidin and has strong bioactivity in anti-cancer and light side-effects, which has been currently applied as a routine anti-cancer agent clinically in China [139]. A recent study has found that norcantharidin loaded in BMSC-derived exosomes have significant antitumour effects and an in-situ homing effect on the tumour sites with no body toxicity in the treatment for hepatocellular carcinoma. Furthermore, BMSC-derived exosomes-loaded norcantharidin can repair damaged liver tissues through promoting cellular proliferation and suppressing liver cell oxidation [140].

### MSC-derived exosomes for radiotherapy damage repair

Despite remarkable advances in radiotherapy techniques, cancer patients often suffer from radiation-induced damage during or after receiving radiotherapy [141]. MSC-derived exosomes have been shown to exert effects on regenerating tissue injuries in different diseases and clinical scenarios models, such as wound healing, cardiovascular disease, and COVID-19 [68]. Multiple lines of evidence have identified that MSC-derived exosomes have promising potential to repair radiation-induced tissue damage by various mechanisms, including promoting cellular proliferation and survival, enhancing angiogenesis, inhibiting inflammation and oxidation, and reducing the pathogenesis of fibrosis [142]. For instance, intravenous delivery of human BMSC-derived extracellular vesicles, including exosomes and microvesicles, can recover the normal counts of peripheral blood cells because the exosomes transfer various miRNAs in mice after receipt of whole-body irradiation [67]. Mechanistically, exosomal miR-221, miR-451, and miR-654-3p can enhance the proliferation of irradiated bone marrow cells, and exosomal miR210-5p, miR-106b-3p and miR-155-5p can prevent the radiation-induced apoptosis of haematopoietic cells [67]. It has also been reported that MSC-derived exosomes can recover radiation-induced haematopoiesis in a mouse model by secreting human haematopoiesis-related cytokines, such as IL-6/8, G-CSF, and MCP-1 [143]. Additionally, MSC-derived exosomes can protect the skin, respiratory system and other systems against radiation-induced damage. BMSC-derived exosomes have been reported to alleviate radiation-induced bone loss in a rat model. This effect is achieved by recovering the cellular proliferative capability, alleviating DNA and oxidative stress damage, and restoring the balance between adipogenesis and osteogenesis of irradiated BMSCs [144]. Radiotherapy-induced lung fibrosis is a common complication of thoracic radiotherapy and strongly limits the increasing radiation doses and influences the cancer patients’ clinical outcomes. Mouse MSC-derived exosomal miR-466f-3p can efficiently relieve radiation-induced lung fibrosis through reversing EMT by inhibiting the AKT/GSK3β signaling pathway via c-MET [145].

## Perspectives and challenges

MSC-derived exosomes are critical mediators of every mentioned category of therapy resistance in various human malignancies. Multiple bioactivators in MSC-derived exosomes may have potential as optimal candidates for noninvasive biomarkers associated with cancer therapy resistance and therapeutic efficacy for cancer patients. Therefore, dynamic monitoring of these bioactivators may be a promising approach to effectively evaluate therapeutic responses, thus enabling clinicians to choose the most suitable therapy strategy for individual patient. Moreover, the identification of exosomes, especially MSC-derived exosomes, as a cell-free material that can exert inherently beneficial therapeutic effects provides a novel avenue for their application in cancer treatment. Bioengineered MSC-derived exosomes can function as delivery vehicles for anticancer agents and diverse synthetic biomolecules, thus exerting excellent anticancer effects. To date, a total of 15 clinical trials employing MSC-derived exosomes as therapeutic agents have been designed or conducted (www.clinicaltrials.gov). Although substantial progress has been achieved in the investigation of MSC-derived exosomes, many crucial unanswered questions and limitations of MSC-derived exosome have slowed down their clinical application, and should be addressed in future studies.

First, standard classifications for MSC-derived exosomes remain elusive. A recent study presented isolation and characterization protocols for six different extracellular vesicle subpopulations from tissues [146]. However, the classification of MSC-derived extracellular vesicles still lacks uniform international standards, and whether previous results have been affected by inconsistent isolation and purification techniques remains unclear. Future studies should distinguish different MSC-derived extracellular vesicle subpopulations and then assess their antitumour activity. Moreover, systematic standards and protocols for the extraction, purification, and storage of MSC-derived exosomes have not been established, which is a problem for the clinical application of MSC-derived exosomes. The use of different isolation and purification methods contribute to different subpopulations of MSC-derived extracellular vesicles with different contents, characteristics and biological functions [66]. Therefore, novel standardized strategies for extraction, purification, and storage techniques that meet good manufacturing practice (GMP) standards need to be developed for MSC-derived exosomes research.

The efficiency of the large-scale production of MSC-derived exosomes also clearly hinders the potential translation of MSC-derived exosomes into clinical practice. Currently, several effective and exosome isolation techniques have been developed, such as the polyethylene glycol (PEG) isolation approach, dielectrophoretic (DEP) separation, and deterministic lateral displacement (DLD) separation, and the combination of two or more isolation techniques presents a plausible strategy for efficient and high purity exosome isolation [147–149]. Nonetheless, combined isolation strategies contribute to high cost and procedure complexity. Developing real-time exosome quantification and analysis strategies and devices is also of great importance for further in-depth investigation and clinical translation of MSC-derived exosomes. It also seems necessary to improve the unsatisfactory efficiency of antitumour cargo loading and delivery in engineered exosomes. Recently, an increasing number of studies have focused on developing artificial exosomes as prominent drug delivery systems. Artificial exosomes possess similar characteristics and therapeutic potential to exosomes with higher pharmaceutical acceptability and the capability of much larger-scale production [150, 151]. Employing MSC-derived exosome mimetics in cancer management is a promising future direction with a wide clinical application view. For instance, hBMSC-derived exosome mimetics mixed with PTX via extrusion can exert promising antitumour effects in breast cancer in vitro and in vivo [152].

Despite their native tumour-homing properties, MSC-derived exosomes can be taken up by different cell types, and achieving promising targeting specificity and to prevent side effects caused by targeting of nontargeted cells is of great importance. Researchers are now exploring and developing novel strategies for attaching specific peptides or ligands to the exosome membrane by surface engineering to improve targeting specificity. Lysosomal-associated membrane protein 2 (Lamp2b), which can be appended with various targeting ligands, has become the most extensively employed exosome membrane protein for exosome surface engineering to improve the selectivity for specific targeted cells [153, 154]. Furthermore, several other exosome membrane proteins, such as the transmembrane protein platelet-derived growth factor receptor (PDGFR), the tetraspanin superfamily members, CD63, CD9, and CD81 (with their two extracellular loops), and lactadherin (with its C1C2 domain) can also be engineered to confer cancer cell targeting specificity [155, 156]. However, novel cancer cell-targeting biomolecules with high binding affinity and targeting specificity are still lacking, and strategies to the problems caused by surface engineering, such as immune activation and host protein deficiency are needed.

Finally, challenges also exist regarding the uncertain biosafety of MSC-derived exosomes in the clinic. There are multiple functional and harmful contents in MSC-derived exosomes; thus, further studies should focus on developing effective and novel engineering strategies to remove or deactivate unwanted and harmful substances in MSC-derived exosomes to ensure the safety of exosomal therapy [157]. In addition, the instability of MSC-derived exosomal contents in recipients is another challenge for the future application of MSC-derived exosomes in cancer management. Previous studies have found that the amount of miRNA cargo secreted by exosomes can be highly influenced by environmental conditions, such as the pH value and hypoxic status of the culture medium [66]. Thus, more efforts should be made to improve the stability of antitumour cargoes in MSC-derived exosomes in the human body. Furthermore, a comprehensive evaluation of the optimal dose, drug distribution, therapeutic routine and biological safety of MSC-derived exosomes in cancer treatment is urgently required. To date, most research focusing on MSC-derived exosomes has been conducted in cellular experiments; thus, the biosafety and efficacy of MSC-derived exosome-associated applications should be examined with long-term monitoring platforms and in vivo models. There is also an urgent need for larger, multicentre, and longer-term studies to achieve the clinical use of MSC-derived exosomes, including clinical trials.

## Conclusions

Overall, we discussed and summarized recent discoveries and research progress related to the roles of MSC-derived exosomes in mediating cancer therapy resistance, and further emphasized the potential of MSC-derived exosomes in clinical applications as novel antitumour agents, which provides a novel direction for reversing therapy resistance and improving prognoses for cancer patients. Due to the key role of MSC-derived exosomes in mediating cancer therapy resistance, further investigations are needed to explore the underlying mechanisms of MSC-derived exosomes in cancer therapy resistance and develop novel MSC-derived exosome-related therapeutic strategies to overcome cancer therapy resistance in the clinic.

## Data Availability

Not applicable.

## Abbreviations

MSCs: mesenchymal stem cells
EMT: epithelial-mesenchymal transition
MSC-derived exosomes: mesenchymal stem cell-derived exosomes
CSCs: cancer stem cells
ncRNAs: noncoding RNAs
MVBs: multivesicular bodies
ILVs: intraluminal vesicles
ESEs: early-sorting endosomes
LSEs: late sorting endosomes
ESCRT: Endosomal-sorting complex required for transport
DUBs: de-ubiquitylating enzymes
VPS4: vacuolar protein sorting 4
ARF6: ADP ribosylation factor 6
SNARE: soluble N-ethylmaleimide-sensitive factor attachment protein receptor
HSPs: heat shock proteins
ssDNA: single-stranded DNA
dsDNA: double stranded DNA
mtDNA: mitochondrial DNA
BMSC: bone marrow mesenchymal stem cell
RBPs: RNA-binding proteins
ADR: adriamycin
5-FU: 5-fluorouracil
MDR: multi-drug resistance
hUCMSCs: MSCs derived from the human umbilical cord
BM2: bone marrow–metastatic human breast cancer cell line
DOX: doxorubicin
AML: acute myeloid leukemia
ADMSC: adipose mesenchymal stem cell
DDP: cisplatin
PTX: paclitaxel
EFGR: epidermal growth factor receptor
TKIs: tyrosine kinase inhibitors
CML: chronic myeloid leukemia
EGFR: epidermal growth factor receptor
PI: proteasome inhibitor
IM: Imatinib
OSCC: oesophageal squamous cell carcinoma
CAR: chimaeric antigen receptor
NK: natural killer
M-MDSCs: monocytic myeloid-derived suppressor cells
OXA: oxaliplatin
ICD: immunogenic cell death
TMZ: temozolomide
WJ-MSCs: Wharton’s jelly-MSCs
siRNA: small interfering RNA
BBB: blood–brain barrier
TRAIL: factor-related apoptosis-inducing ligand
GEMP: gemcitabine monophosphate
PEG: polyethylene glycol (PEG)
DEP: dielectrophoretic
DLD: deterministic lateral displacement
Lamp2b: lysosomal-associated membrane protein 2
PDGFR: platelet-derived growth factor receptor
